# Effects of Conjugated Linoleic Acid on Antioxidant and Immune Functions of Perinatal Ewes and Lambs

**DOI:** 10.3390/ani16213444

**Published:** 2026-10-31

**Authors:** Minghui Liu, Terigele Wang, Hang Zhang, Ni Dan, Zheming Ren, Wenqi Liang, Pengfei Sun

**Affiliations:** 1 College of Animal Science and Technology, Inner Mongolia Minzu University, Tongliao 028000, China; 18419336807@163.com (M.L.);; 2 Tongliao Gadasu Breeding Stock Center, Tongliao 028015, China; 3 College of Life Sciences and Food Engineering, Inner Mongolia Minzu University, Tongliao 028000, China

**Keywords:** conjugated linoleic acid, perinatal ewe, lamb, antioxidant, immunity

## Abstract

This study evaluated the effects of dietary conjugated linoleic acid (CLA) on serum metabolites, antioxidant capacity, and immune function of transition ewes, as well as on growth performance, serum biochemical indices, and hormone levels of their suckling lambs. Forty healthy Ujumqin ewes (body weight 56.62 ± 5.30 kg) were randomly assigned to four groups (*n* = 10/group) at 28 d before expected parturition: control (basal diet), CLA-1 (basal diet + 10 g/d CLA), CLA-2 (basal diet + 20 g/d CLA), and CLA-3 (basal diet + 30 g/d CLA). Blood samples were collected from ewes at 21 d prepartum, at delivery, and at 21 d postpartum, and from lambs at 7 and 42 d postpartum. Serum metabolites, antioxidant enzymes, and inflammatory cytokines were measured. In ewes, CLA supplementation did not significantly affect serum AST, β-HB, GLU, TG, NEFA, SOD, GPX, MDA, or T-AOC (*p* > 0.05), although β-HB, GLU, SOD, GPX, and MDA changed with time. However, CLA markedly decreased serum TNF-α, IL-8, IL-6, and IL-1β (*p* < 0.05). In lambs, maternal CLA did not affect birth or final body weight. Maternal CLA decreased serum GH and the GH/IGF-1 ratio and increased IGF-1 (*p* < 0.05), especially at 30 g/d. Serum SOD, GPX, and MDA did not differ among groups, whereas T-AOC showed a significant group × time interaction (*p* = 0.004). Maternal CLA also decreased serum TNF-α, IL-8, IL-6, and IL-1β in lambs (*p* < 0.05). In conclusion, perinatal CLA supplementation did not significantly improve most energy metabolic or antioxidant enzyme indices under these conditions, but it alleviated excessive inflammatory responses in ewes and suckling lambs and activated the GH-IGF-1 axis in lambs. The 30 g/d CLA dose showed the strongest immunomodulatory and endocrine effects. These findings suggest that CLA may serve mainly as an immunomodulatory strategy in perinatal sheep, and dose optimization warrants further study.

## Simple Summary

Feeding conjugated linoleic acid (CLA) to ewes during the transition period did not significantly alter most serum energy metabolites or antioxidant enzyme indices, but it reduced excessive inflammatory responses in both ewes and their suckling lambs. Maternal CLA supplementation also decreased growth hormone and increased IGF-1 in lambs, indicating modulation of the somatotropic axis. These findings support CLA as a potential nutritional strategy for immunomodulation and endocrine regulation in perinatal sheep.

## 1. Introduction

The perinatal period, typically defined as 21 d prepartum to 21 d postpartum, represents the most challenging phase in the ewe’s production cycle, with profound physiological and metabolic demands [1]. During late gestation, fetal growth accelerates, dramatically increasing nutrient requirements, while concurrent uterine compression and hormonal changes often reduce dry matter intake, leading to a negative energy balance (NEB) [2]. Negative energy balance is characterized by excessive body fat mobilization, reflected by elevated serum nonesterified fatty acids (NEFA) and β-hydroxybutyrate (β-HB) [3]. Ewes with multiple pregnancies are particularly susceptible to pregnancy toxemia due to limited hepatic gluconeogenic capacity [4]. High NEFA concentrations not only indicate lipid mobilization but also act as pro-inflammatory signals, activate oxidative pathways, and generate reactive oxygen species (ROS) through hepatic β-oxidation, thereby inducing oxidative stress and immunosuppression [5]. Such metabolic disturbances increase the risk of pregnancy toxemia and postpartum ketosis, and severely impair fetal development, resulting in low birth weight and reduced lamb vigor, which compromise survival and later performance [6]. Therefore, nutritional strategies to alleviate NEB, oxidative stress, and inflammation during the perinatal period are of great importance for the sheep industry [7].

Conjugated linoleic acid (CLA) is a group of positional and geometric isomers of linoleic acid, naturally found in ruminant products, and has attracted considerable attention for its roles in lipid metabolism, immune modulation, and oxidative stress mitigation [8]. CLA can activate peroxisome proliferator-activated receptor γ (PPARγ) signaling, affecting adipocyte differentiation and apoptosis, thereby reducing body fat deposition [9]. In a sheep mastitis model, CLA was shown to upregulate antioxidant enzymes (SOD, nuclear factor erythroid 2-related factor 2, heme oxygenase-1) via the PPARγ-UCP2 pathway, while downregulating pro-inflammatory cytokines (IL-1β, IL-6, and nuclear factor κB) [10]. In vitro studies also demonstrated that CLA improves redox status in bovine mammary epithelial cells and modulates inflammatory functions in bovine monocytes [11,12]. These findings support the biological basis for using CLA as an antioxidant and immunomodulatory agent in ruminants. However, most CLA research in ruminants has focused on dairy cows, particularly on energy metabolism and milk fat synthesis. Veshkini et al. [13] suggested that CLA-induced milk fat depression spares metabolic energy, alleviating NEB during early lactation. Abomasal infusion of CLA in transition cows [14] affected oxidative stress markers in plasma, erythrocytes, and liver, with time-dependent effects on immunohematological parameters. In sheep, Kiarash et al. [15] examined energy metabolism and liver function in perinatal ewes but focused on different fat sources rather than CLA. Thus, systematic studies on CLA in perinatal ewes are scarce, lacking comprehensive evaluation of dynamic changes in energy metabolism, oxidative stress, and immunity across the transition period.

Maternal nutrition during the perinatal period profoundly influences offspring development [16]. Prepartum supplementation with polyunsaturated fatty acids can alter colostrum fatty acid profiles, increasing CLA content and affecting passive immunity and inflammatory responses in lambs [17,18]. Maternal supplementation with essential fatty acids and CLA during late gestation and early lactation affected calf muscle and adipose tissue development [19]; maternal linseed supplementation significantly altered CLA isomer concentrations in lamb muscle and liver through uterine and lactational transfer [20]. However, direct evidence on the effects of maternal CLA supplementation on growth and serum hormone profiles of suckling lambs remains limited.

Therefore, this experiment used Ujumqin perinatal ewes and their lambs to investigate the effects of different levels of rumen-protected CLA on maternal serum protein and energy metabolism, antioxidant capacity, and immune function at prepartum, parturition, and postpartum stages, as well as on lamb growth performance, serum metabolites, and growth-related hormones. The goal was to provide a scientific basis for using CLA as a safe and effective feed additive for perinatal ewes.

## 2. Materials and Methods

### 2.1. Animals and Experimental Design

This experiment was approved by the Medical and Life Sciences Ethics Committee of Inner Mongolia Minzu University (approval No. NMD-DW-2025-11-75). Forty at 28 d before the expected parturition healthy Ujumqin ewes (body weight = 56.62 ± 5.30 kg) were randomly assigned to 4 groups (*n* = 10 per group): control group (CN, fed a basal diet), CLA-1 group (fed the basal diet supplemented with 10 g/d of CLA), CLA-2 group (fed the basal diet supplemented with 20 g/d of CLA), and CLA-3 group (fed the basal diet supplemented with 30 g/d of CLA). The ingredient composition and nutrient levels of the basal diet are presented in Table 1.

The CLA product (Qingdao Aohai Biotechnology Co., Ltd., Qingdao, China) had a purity of 93.8%, containing 45.7% cis-9, trans-11 CLA, 44.8% trans-10, cis-12 CLA, and 3.3% other CLA isomers. The product was rumen-protected by Hangzhou King Techina Feed Co., Ltd. (Hangzhou, China), with a post-protection rumen bypass rate of 74.8% and a measured CLA content of 39.6%.

### 2.2. Feeding Management

The experiment lasted 70 d in total, including a 7 d adaptation period during which all ewes were fed the basal diet. During the 63 d formal experimental period (from 21 d prepartum to 42 d postpartum), ewes in the control group were fed a total mixed ration (TMR) diet prepared using a mixer (Shandong Xinshengtai Machinery Manufacturing Co., Ltd., Jining, China), whereas ewes in the treatment groups were fed the TMR diet with the rumen-protected CLA product mixed in individually before feeding. The supplementation was continued until 42 d postpartum to ensure adequate and high-quality milk supply for the suckling lambs. All ewes had free access to water. Routine disinfection and vaccination procedures were carried out in accordance with standard farm protocols.

### 2.3. Sample Collection and Measurements

Lamb Growth Performance—Birth weight (BW) of lambs was recorded on the day of parturition, and ear tags were applied. All lambs were weighed at 42 d of age to record the final body weight (FBW). Average daily gain (ADG) was calculated from birth to 42 d of age using the formula: ADG = (FBW − BW)/42 days.

Blood sampling and biochemical analyses—Blood samples (10 mL) were collected from the jugular vein of ewes at 21 d prepartum, on the day of parturition, and at 21 d postpartum, as well as from lambs at 7 and 42 d of age. At the beginning of the trial, six ewes per treatment were randomly selected from the 10 ewes in each group to serve as representative subjects for blood collection. Throughout the trial, all ewes were housed and fed individually in separate pens; thus, the individual ewe was considered the experimental unit. The exact same six ewes were sampled at all three time points. The sampled lambs were the exact direct offspring of the six maternal ewes used for blood sampling. All ewes gave birth to a single lamb, ensuring a strict one-to-one maternal-offspring pairing. Because no ewe had multiple lambs included in the study, the lambs were considered completely independent experimental units, and maternal identity did not need to be included as a random effect. The sex distribution of the sampled lambs was recorded, consisting of 11 males and 13 females. Blood samples were centrifuged at 1800× *g* for 10 min; serum was separated, aliquoted into 1.5 mL centrifuge tubes, and stored at −20 °C until analysis.

Serum concentrations of albumin (ALB), aspartate aminotransferase (AST), total protein (TP), cholesterol (CHO), β-hydroxybutyrate (β-HB), glucose (GLU-YA), high-density lipoprotein cholesterol (HDL-C), low-density lipoprotein cholesterol (LDL-C), and triglycerides (TG) were determined using an automatic biochemical analyzer (BK-200, Biobase, Jinan, China) with commercial kits (Biobase, Jinan, China). Serum antioxidant parameters, including total antioxidant capacity (T-AOC), superoxide dismutase (SOD), glutathione peroxidase (GPX), and malondialdehyde (MDA), were determined using a microplate reader (Infinite M Nano, Tecan, Austria) with kits purchased from Beijing Solarbio Science & Technology Co., Ltd. (Beijing, China). Serum concentrations of immune-related cytokines, including tumor necrosis factor-α (TNF-α), interleukin-8 (IL-8), IL-6, and IL-1β, as well as growth hormone (GH) and insulin-like growth factor-1 (IGF-1), were determined by ELISA using kits from Shanghai Ruixin Biotechnology Co., Ltd. (Shanghai, China).

### 2.4. Statistical Analysis

Statistical analyses were performed using SPSS 26.0. Values are expressed as mean ± standard error of the mean (SEM). For ewe data, repeated-measures analysis of covariance (RM-ANCOVA) was used, with treatment as a fixed effect and time as the within-subject repeated factor; data collected 21 d prepartum were included as a covariate to control for individual baseline differences. The time main effect, the treatment main effect, and the time × treatment interaction were analyzed. Growth performance was analyzed using one-way ANOVA. For lamb data, repeated-measures ANOVA (RM-ANOVA) was used to analyze the time main effect, the treatment main effect, and the time × treatment interaction. Mauchly’s test was used to assess sphericity; when the sphericity assumption was violated (*p* < 0.05), the Greenhouse-Geisser correction was applied to adjust the degrees of freedom. When the overall effect was significant, Bonferroni correction was used for post hoc multiple comparisons. Differences were considered significant at *p* < 0.05 and a trend toward significance at 0.05 ≤ *p* < 0.10.

## 3. Result

### 3.1. Effects of Conjugated Linoleic Acid on Serum Protein and Energy Metabolism-Related Indicators in Perinatal Ewes

As shown in Table 2, after adjustment for baseline values, serum β-HB was higher at 21 d postpartum than on the day of parturition (*p* = 0.011), whereas serum GLU was lower at 21 d postpartum than on the day of parturition (*p* = 0.022). No significant differences among CN, CLA-1, CLA-2, and CLA-3 were observed for AST, β-HB, GLU, TG, or NEFA on the day of parturition or at 21 d postpartum. No significant differences in AST, TG, or NEFA were observed between the day of parturition and 21 d postpartum.

### 3.2. Effects of Conjugated Linoleic Acid on Serum Antioxidant Indicators in Perinatal Ewes

As shown in Table 3, after adjustment for baseline values, serum SOD (*p* < 0.001) and MDA (*p* = 0.006) were lower at 21 d postpartum than on the day of parturition, whereas serum GPX was higher at 21 d postpartum than on the day of parturition (*p* < 0.001). No significant differences among treatments were observed for SOD, GPX, MDA, or TAOC on the day of parturition or at 21 d postpartum. No significant difference in TAOC was observed between the day of parturition and 21 d postpartum.

### 3.3. Effects of Conjugated Linoleic Acid on Serum Immune Indicators in Perinatal Ewes

As shown in Table 4, serum TNF-α, IL-8, IL-6, and IL-1β differed among the four groups (*p* < 0.01). The main effect of time was significant for serum IL-8 (*p* = 0.026), but no significant pairwise differences were detected between the day of parturition and 21 d postpartum. At the day of parturition and at 21 d postpartum, TNF-α was higher in CN than in CLA-2 and CLA-3, and CLA-3 was lower than CN, CLA-1, and CLA-2. IL-8 was higher in CN than in CLA-2 and CLA-3 at both time points, whereas CLA-1 did not differ significantly from CN, CLA-2, or CLA-3. IL-6 was higher in CN than in CLA treatments at both time points, with no significant differences among the CLA treatments. IL-1β was higher in CN than in CLA-3 at both time points, whereas CLA-1 and CLA-2 did not differ significantly from CN or CLA-3. No significant differences between the day of parturition and 21 d postpartum were observed for TNF-α, IL-8, IL-6, or IL-1β.

### 3.4. Effects of Conjugated Linoleic Acid on Growth Performance of Lambs

As shown in Table 5, no significant differences were observed in initial body weight (IBW), final body weight (FBW), or average daily gain (ADG) among the four groups (*p* > 0.05).

### 3.5. Effects of Conjugated Linoleic Acid on Serum Protein and Energy Metabolism-Related Indicators and Hormones in Lambs

As shown in Table 6, serum GLU was higher at 7 d of age than at 42 d of age (*p* = 0.002), whereas no significant differences were observed among the four groups. Serum TG differed among the four groups (*p* = 0.012) and was higher at 7 d of age than at 42 d of age (*p* < 0.001); no significant group × time interaction was observed. At 7 d of age, TG was higher in CN than in CLA-2 and CLA-3, whereas CLA-1 did not differ significantly from CN, CLA-2, or CLA-3. At 42 d of age, TG was higher in CN than in CLA-1 and CLA-2, whereas CLA-3 did not differ significantly from CN, CLA-1, or CLA-2. In all groups, TG was lower at 42 d of age than at 7 d of age. No significant differences among groups or between time points were observed for AST, β-HB, or NEFA.

As shown in Table 7, serum GH differed among the four groups and between 7 and 42 d of age (*p* < 0.05); at both time points, GH was higher in CN and CLA-1 than in CLA-2 and CLA-3, and higher in CLA-2 than in CLA-3. IGF-1 differed among groups (*p* < 0.001), with higher concentrations in CLA-3 than in CN and no significant differences among the other groups. The GH/IGF-1 ratio differed among groups (*p* < 0.001); CN, CLA-1, and CLA-2 were higher than CLA-3, and CN was higher than CLA-2, whereas CN vs. CLA-1 and CLA-1 vs. CLA-2 did not differ significantly. No significant group × time interaction was observed for any indicator, and no significant time effect was observed for IGF-1 or the GH/IGF-1 ratio.

### 3.6. Effects of Conjugated Linoleic Acid on Serum Antioxidant and Immune Indicators in Lambs

As shown in Table 8, serum GPX (*p* = 0.001) and MDA (*p* < 0.001) were higher at 42 d of age than at 7 d of age in lambs. No significant differences among CN, CLA-1, CLA-2, and CLA-3 were observed for SOD, GPX, or MDA at 7 or 42 d of age in lambs. No significant difference in SOD was observed between 7 and 42 d of age in lambs. A significant treatment × time interaction was observed for TAOC (*p* = 0.004); at 7 d of age in lambs, TAOC was higher in CLA-2 and CLA-3 than in CN and CLA-1 in the serum of lambs, whereas no significant differences among groups were observed at 42 d of age. TAOC was lower at 42 d than at 7 d in CLA-2 and CLA-3 in the serum of lambs, whereas no significant difference between 7 and 42 d was observed in CN or CLA-1 in the serum of lambs.

As shown in Table 9, the TNF-α, IL-8, IL-6, and IL-1β were different among the four groups (*p* < 0.001) in the serum of lambs. Across both sampling times, TNF-α was higher in CN and CLA-1 than in CLA-2 and CLA-3. IL-8 was higher in CN than in CLA-1, CLA-2, and CLA-3, and higher in CLA-1 than in CLA-3, whereas CLA-1 vs. CLA-2 and CLA-2 vs. CLA-3 did not differ significantly in the serum of lambs. IL-6 was higher in CN than in CLA-2 and CLA-3, and higher in CLA-1 and CLA-2 than in CLA-3; CN vs. CLA-1 and CLA-1 vs. CLA-2 did not differ significantly in the serum of lambs. IL-1β was higher in CN than in CLA-2 and CLA-3, whereas CLA-1 did not differ significantly from CN, CLA-2, or CLA-3 in the serum of lambs. Serum IL-6 (*p* = 0.011) and IL-1β (*p* < 0.001) were higher at 7 d of age than at 42 d of age in lambs. No significant time effect was observed for TNF-α or IL-8, and no significant group × time interaction was observed for any indicator.

## 4. Discussion

### 4.1. Effects of CLA on Serum Metabolites, Antioxidant Capacity, and Immune Function in Perinatal Ewes

Energy metabolism. Perinatal ewes often experience negative energy balance (NEB) due to reduced dry matter intake, leading to excessive body fat mobilization, which is reflected by elevated serum NEFA and β-HB and decreased GLU [2,13]. Similar metabolic challenges and their systemic impacts have also been extensively documented in transition dairy cows [21]. NEFA is a direct product of lipolysis, whereas β-HB is the end product of NEFA oxidation in the liver; together, they provide a comprehensive assessment of fat mobilization [13,14]. In the present study, dietary CLA supplementation did not significantly alter serum AST, β-HB, GLU, TG, or NEFA concentrations among groups. All groups showed similar time-dependent changes, with β-HB increasing and GLU decreasing from parturition to 21 d postpartum, and no significant group × time interaction was detected. These results indicate that the postpartum metabolic challenge occurred irrespective of CLA treatment and that CLA did not markedly modify the temporal pattern of energy metabolite changes. Therefore, under the present conditions, CLA did not significantly improve the energy metabolic status of perinatal ewes. The absence of significant treatment effects may be related to the doses tested, the bioavailability of the rumen-protected product, the use of prepartum values as a covariate, or the limited number of ewes sampled for blood. It should also be noted that only serum metabolites were measured; hepatic gluconeogenic capacity, adipose tissue lipolysis, and PPARγ signaling were not assessed, and these tissue-level changes may not be fully reflected in peripheral blood. Further studies with higher doses, different CLA formulations, tissue-level measurements, or larger sample sizes are needed to determine whether CLA can produce measurable improvements in energy metabolism in perinatal ewes.

Antioxidant status. Maintaining redox homeostasis is crucial for animal health [22]. SOD and GPX are core enzymes that scavenge free radicals and alleviate oxidative damage [23], T-AOC reflects the comprehensive antioxidant reserve capacity of the body [24], and MDA is the end product of lipid peroxidation whose decrease indicates alleviation of lipid peroxidation damage [25]. In dairy cows, elevated serum MDA has been closely associated with subclinical ketosis and severe metabolic stress [26], further supporting its role as a sensitive marker for oxidative damage. In non-ruminant or cell models, CLA can upregulate antioxidant enzyme gene expression through activation of the Nrf2-ARE signaling pathway [27]. In the present study, however, serum SOD, GPX, MDA, and T-AOC did not differ significantly among groups. Significant time effects were observed for SOD, GPX, and MDA, indicating that the redox status of ewes changed dynamically across the transition period, but these changes were not modified by CLA supplementation. Although some CLA-supplemented groups showed numerically lower MDA or higher T-AOC, these numerical differences did not reach statistical significance and should not be interpreted as treatment effects. Thus, the present results do not support a significant enhancing effect of CLA on the endogenous antioxidant enzyme system of perinatal ewes at the doses tested. The discrepancy with previous studies may reflect dose-, tissue-, or time-specific effects of CLA, or the possibility that antioxidant responses occur earlier in the postpartum period and were not captured by the present sampling schedule. In addition, serum enzyme activities reflect only systemic antioxidant capacity, whereas CLA may exert local antioxidant effects in the liver or mammary gland that are not detectable in peripheral blood. More frequent sampling, tissue-level antioxidant measurements, and higher doses will be needed to clarify the antioxidant potential of CLA in perinatal ewes.

Immune function. In many pathological conditions, increased production of TNF-α aggravates inflammatory responses, ultimately resulting in tissue damage and immunosuppression [28]. In contrast to the energy metabolic and antioxidant results, dietary CLA significantly reduced serum pro-inflammatory cytokine concentrations in perinatal ewes. TNF-α and IL-8 were lower in the medium- and high-dose CLA groups than in the control group, with the lowest TNF-α concentration in the high-dose group. IL-6 was lower in all CLA-supplemented groups than in the control group, whereas IL-1β was lower in the high-dose CLA group than in the control group. The different dose responsiveness among cytokines suggests that IL-6 may be more sensitive to CLA, whereas TNF-α, IL-8, and IL-1β require higher doses; this may reflect differences in cell sources, signaling pathways, or transcriptional regulation among cytokines, although the exact mechanism remains unclear. Taken together, these results indicate that CLA can effectively inhibit excessive inflammatory responses in perinatal ewes under the present conditions. The mechanism may involve the PPARγ-NF-κB signaling pathway [29]. Studies in sheep have shown that CLA can reduce the expression of IL-1β, IL-6, and NF-κB through upregulation of the PPARG-UCP2 pathway [10], and in sheep ruminal epithelial cells, CLA downregulated TNF-α and IL-6 expression [30]. However, because PPARγ, NF-κB, and other signaling molecules were not measured in the present study, this mechanism remains speculative. Moreover, since CLA did not significantly alter serum antioxidant enzyme activities, the anti-inflammatory effect may not depend primarily on systemic antioxidant capacity but rather on direct modulation of inflammatory signaling pathways in immune cells. This hypothesis warrants further molecular-level investigation. Finally, the reduction in inflammatory cytokines may help alleviate perinatal immunosuppression and metabolic disturbance, but its effects on ewe health, lactation performance, and lamb growth still need to be verified in production trials.

### 4.2. Maternal CLA Affects Lamb Growth Performance and Hormonal Profile

Growth performance. The GH-IGF-1 axis is a core endocrine regulator of animal growth, and IGF-1 promotes cell proliferation and skeletal muscle growth [31]. In the present study, the macroscopic growth performance (IBW, FBW, and ADG) of the suckling lambs remained statistically comparable among all groups. However, the serum IGF-1 levels were elevated in the CLA-supplemented groups. This suggests that CLA activated the anabolic potential at the tissue level; the lack of significant weight differences may be because the 42-day suckling period was too short for these molecular changes to fully manifest as macroscopic advantages. Furthermore, the relatively stable serum energy metabolites (AST, β-HB, and GLU) across groups indicated that the lambs maintained a normal metabolic status. Previous studies have demonstrated that maternal fatty acid nutrition during late gestation and early lactation can program offspring tissue development [19,32]. Combined with previous evidence that maternal nutrition affects offspring through blood circulation [33], we speculate that maternal CLA may modulate the offspring’s endocrine system. Overall, maternal CLA may exert a long-term programming effect on lamb growth, which warrants verification through extended observation periods.

Hormonal profile. Growth hormone and IGF-1 form a closed negative feedback loop: GH stimulates hepatic synthesis and secretion of IGF-1, while elevated IGF-1 in turn inhibits pituitary GH release [34]. In the present study, maternal CLA supplementation significantly altered this axis in a dose-dependent manner. Specifically, the high-dose CLA group exhibited the most pronounced endocrine shift, characterized by persistently elevated serum IGF-1, decreased GH levels, and the lowest GH/IGF-1 ratio across both sampling times. This endocrine pattern is consistent with classical negative feedback regulation, indicating that maternal CLA shifted the metabolic status of the lambs toward a strong anabolic state. These findings provide a mechanistic explanation for the tissue-level anabolic potential observed in the Growth performance section, despite the lack of significant macroscopic weight gain within the short suckling period.

### 4.3. Effects of Maternal CLA on Antioxidant and Immune Indicators in Suckling Lambs

Antioxidant status. SOD and GPX are core antioxidant enzymes, T-AOC reflects the total antioxidant reserve, and MDA reflects lipid peroxidation [23,24,25]. In calves, maternal EFA and CLA supplementation during late pregnancy and early lactation can affect the oxidative and antioxidant status of neonatal plasma [33]. In the present study, serum SOD, GPX, and MDA did not differ significantly among groups at either 7 or 42 d of age. Significant time effects were observed for GPX and MDA, which were higher at 42 d than at 7 d of age, indicating that the antioxidant status of lambs changed with age, but these changes were not modified by maternal CLA supplementation. The only antioxidant indicator that showed a significant treatment effect was T-AOC. A significant group × time interaction was observed for T-AOC: at 7 d of age, T-AOC was higher in the medium- and high-dose CLA groups than in the control and low-dose groups, whereas no significant differences among groups were observed at 42 d of age. T-AOC decreased from 7 to 42 d of age in the medium- and high-dose CLA groups, whereas no significant change was observed in the control or low-dose group. These results suggest that maternal CLA supplementation may enhance the early postnatal total antioxidant reserve of suckling lambs, but this effect appears to be transient and was not accompanied by changes in SOD, GPX, or MDA. The lack of significant effects on SOD, GPX, and MDA may be related to the limited number of lambs sampled, the timing of blood collection, or the possibility that enzyme activities are tightly regulated and less responsive to maternal CLA than T-AOC. Therefore, the present results should not be interpreted as a general enhancement of lamb antioxidant capacity by maternal CLA; rather, they suggest a selective and transient effect on T-AOC that warrants further investigation.

Immune function. Immunoglobulins and cytokines are commonly used to evaluate immune function, and sIgA is a major immunoglobulin in the intestinal mucosa that interacts with gut microorganisms to enhance mucosal immunity and barrier function [35]. In the present study, maternal CLA supplementation significantly reduced serum pro-inflammatory cytokine concentrations in lambs. TNF-α was lower in the medium- and high-dose CLA groups than in the control and low-dose groups at both sampling times. IL-8 was lower in all CLA-supplemented groups than in the control group, and lower in the high-dose group than in the low-dose group. IL-6 was lower in the medium- and high-dose CLA groups than in the control group, and lower in the high-dose group than in the low- and medium-dose groups. IL-1β was lower in the medium- and high-dose CLA groups than in the control group. Serum IL-6 and IL-1β were higher at 7 d than at 42 d of age, whereas no significant time effects were observed for TNF-α or IL-8, and no significant group × time interaction was observed for any cytokine. These results indicate that maternal CLA supplementation can suppress excessive inflammatory responses in suckling lambs, and the effect appears to persist until 42 d of age for TNF-α and IL-8. The mechanism may involve the transfer of active CLA components through colostrum and milk, activating the PPARγ signaling pathway in lambs and inhibiting NF-κB-mediated transcription and release of pro-inflammatory cytokines. However, PPARγ and NF-κB were not measured, and the CLA content of colostrum and milk was not determined; therefore, this mechanism remains speculative. Studies have shown that maternal fatty acid supplementation can affect the development and maturation of the offspring immune system through colostrum and transitional milk intake [35], which is consistent with the changes in lamb immune indicators observed here. Combined with the transient enhancement of T-AOC, these findings suggest that maternal CLA supplementation is associated with enhanced anti-inflammatory capacity in suckling lambs, providing favorable conditions for early growth and development. Nevertheless, only serum cytokines were measured, and immune cell function, immunoglobulin levels, and disease incidence were not assessed; therefore, the practical significance of these changes for lamb health and survival remains to be determined.

## 5. Conclusions

Perinatal CLA supplementation did not significantly improve most serum energy metabolic indices or systemic antioxidant enzyme activities in ewes, but it alleviated excessive inflammatory responses. Maternal CLA supplementation also modulated the GH-IGF-1 axis in suckling lambs and reduced their serum pro-inflammatory cytokine concentrations, suggesting maternal-offspring nutrient transfer, although the specific route remains undetermined. Under the conditions of this experiment, 30 g/d CLA showed the strongest immunomodulatory and endocrine effects and was the most effective dose; whether higher doses produce saturation effects and how growth responds still require further verification.

## Figures and Tables

**Table 1:** Ingredient and nutrient composition of the basal diet (dry matter basis).

Ingredients	Content (%)	Nutrient Levels ^2^	
Cured hay	37	ME (MJ/kg)	10.02
Silage	30	CP (%)	11.86
Corn flour	14.5	EE (%)	3.95
Wheat bran	3	NDF (%)	43.29
Soybean meal	6.5	ADF (%)	26.57
DDGS	3	Ca (%)	0.81
Yeast culture	0.2	P (%)	0.38
Rumen-protected fat	1.5		
Molasses	2		
Calcium hydrogen phosphate	0.5		
Calcium carbonate	0.7		
Sodium bicarbonate	0.3		
NaCl	0.3		
Premix ^1^	0.5		
Total	100		

^1^ 800. IU; vitamin D, 160,000 IU; vitamin E, 5000 mg; Cu, 2000 mg; Fe, 8000 mg; Mn, 8000 mg; Zn, 9000 mg; I, 80 mg; Se, 40 mg; Co, 20 mg. ^2^ 816. Nutrient Requirements of Meat Sheep, and all other values were determined by analysis.

**Table 2:** Effects of CLA on serum protein and energy metabolism indices in perinatal ewes.

Item	Time	CN	CLA-1	CLA-2	CLA-3	*p*-Value			
						Group	Time	Interaction	Baseline
AST/(U/L)	d_0_	146 ± 11	145 ± 10	142 ± 10	118 ± 10	0.485	0.789	0.412	0.187
	d_21_	167 ± 11	165 ± 10	159 ± 10	158 ± 11				
β-HB/(mmol/L)	d_0_	0.55 ± 0.04 ^B^	0.50 ± 0.04 ^B^	0.44 ± 0.04 ^B^	0.43 ± 0.04 ^B^	0.803	0.011	0.143	0.373
	d_21_	0.76 ± 0.10 ^A^	0.67 ± 0.10 ^A^	0.62 ± 0.10 ^A^	0.50 ± 0.10 ^A^				
GLU/(mmol/L)	d_0_	4.53 ± 0.64 ^A^	5.16 ± 0.63 ^A^	5.45 ± 0.63 ^A^	5.55 ± 0.65 ^A^	0.871	0.022	0.203	0.058
	d_21_	3.91 ± 0.37 ^B^	4.45 ± 0.36 ^B^	4.61 ± 0.37 ^B^	4.89 ± 0.36 ^B^				
TG/(mmol/L)	d_0_	0.28 ± 0.04	0.26 ± 0.04	0.23 ± 0.04	0.21 ± 0.04	0.828	0.357	0.353	0.627
	d_21_	0.31 ± 0.05	0.31 ± 0.05	0.26 ± 0.05	0.21 ± 0.05				
NEFA/(mmol/L)	d_0_	0.62 ± 0.08	0.60 ± 0.08	0.57 ± 0.08	0.49 ± 0.08	0.726	0.386	0.367	0.599
	d_21_	0.66 ± 0.10	0.57 ± 0.10	0.54 ± 0.10	0.46 ± 0.10				

Note: Different uppercase letters in the same column indicate significant differences between time points within the same treatment group (*p* < 0.05). The same letter or no letter indicates no significant difference (*p* > 0.05). *P*-group, *P*-time, and *P*-interaction denote the main effects of treatment group, time, and group × time interaction, respectively.

**Table 3:** Effects of CLA on Serum Antioxidant Indicators in Perinatal Ewes.

Item	Time	CN	CLA-1	CLA-2	CLA-3	*p*-Value			
						Group	Time	Interaction	Baseline
SOD/(U/mL)	d_0_	4.18 ± 1.15 ^A^	5.66 ± 1.05 ^A^	7.36 ± 1.08 ^A^	8.45 ± 1.22 ^A^	0.126	<0.001	0.094	0.242
	d_21_	2.14 ± 0.41 ^B^	2.73 ± 0.40 ^B^	2.80 ± 0.44 ^B^	3.15 ± 0.46 ^B^				
GPX/(U/mL)	d_0_	54.6 ± 6.7 ^B^	55.5 ± 6.5 ^B^	63.0 ± 6.6 ^B^	63.8 ± 7.0 ^B^	0.772	<0.001	0.539	0.843
	d_21_	71.6 ± 2.5 ^A^	75.4 ± 2.5 ^A^	75.6 ± 2.6 ^A^	77.0 ± 2.7 ^A^				
MDA/(nmol/mL)	d_0_	2.39 ± 0.32 ^A^	2.11 ± 0.35 ^A^	1.67 ± 0.32 ^A^	1.41 ± 0.32 ^A^	0.296	0.006	0.346	0.346
	d_21_	1.66 ± 0.29 ^B^	1.35 ± 0.27 ^B^	1.24 ± 0.27 ^B^	1.22 ± 0.27 ^B^				
T-AOC/(U/mL)	d_0_	0.57 ± 0.12	0.62 ± 0.13	0.69 ± 0.14	0.97 ± 0.15	0.193	0.273	0.365	0.392
	d_21_	0.61 ± 0.05	0.61 ± 0.05	0.68 ± 0.05	0.68 ± 0.06				

Note: Different uppercase letters in the same column indicate significant differences between time points within the same treatment group (*p* < 0.05). The same letter or no letter indicates no significant difference (*p* > 0.05). *P*-group, *P*-time, and *P*-interaction denote the main effects of treatment group, time, and group × time interaction, respectively.

**Table 4:** Effects of CLA on serum immune indicators in perinatal ewes.

Item	Time	CN	CLA-1	CLA-2	CLA-3	*p*-Value			
						Group	Time	Interaction	Baseline
TNF-α/(ng/g)	d_0_	171 ± 12 ^a^	156 ± 12 ^ab^	126 ± 12 ^b^	100 ± 13 ^c^	<0.001	0.550	0.948	0.125
	d_21_	163 ± 11 ^a^	150 ± 11 ^ab^	129 ± 11 ^b^	91 ± 11 ^c^				
IL-8/(ng/g)	d_0_	673 ± 31 ^a^	530 ± 24 ^ab^	490 ± 24 ^b^	386 ± 31 ^b^	0.002	0.026	0.550	0.960
	d_21_	552 ± 50 ^a^	484 ± 37 ^ab^	430 ± 37 ^b^	406 ± 49 ^b^				
IL-6/(ng/g)	d_0_	139 ± 9 ^a^	116 ± 9 ^b^	100 ± 8 ^b^	98 ± 12 ^b^	0.001	0.516	0.816	0.965
	d_21_	149 ± 9 ^a^	123 ± 9 ^b^	107 ± 8 ^b^	88 ± 12 ^b^				
IL-1β/(ng/g)	d_0_	70.0 ± 3.7 ^a^	56.7 ± 2.6 ^ab^	54.1 ± 2.6 ^ab^	47.2 ± 3.0 ^b^	<0.001	0.311	0.299	0.220
	d_21_	63.1 ± 3.5 ^a^	59.2 ± 2.4 ^ab^	56.6 ± 2.5 ^ab^	44.9 ± 2.9 ^b^				

Note: Different lowercase letters in the same row indicate significant differences among treatment groups at the same time point (*p* < 0.05). Different uppercase letters in the same column indicate significant differences between time points within the same treatment group (*p* < 0.05). The same letter or no letter indicates no significant difference (*p* > 0.05). Uppercase and lowercase letters are independent. *P*-group, *P*-time, and *P*-interaction denote the main effects of treatment group, time, and group × time interaction, respectively.

**Table 5:** Effects of CLA on growth performance of lambs.

Items	Control Group	CLA-1 Group	CLA-2 Group	CLA-3 Group	*p*-Value
IBW/kg	4.67 ± 0.14	4.80 ± 0.16	4.67 ± 0.17	4.72 ± 0.15	0.921
FBW/kg	14.40 ± 0.25	14.73 ± 0.49	13.73 ± 0.56	14.63 ± 0.36	0.381
ADG/(kg/d)	0.230 ± 0.006	0.237 ± 0.015	0.217 ± 0.011	0.235 ± 0.012	0.598

**Table 6:** Effects of conjugated linoleic acid on serum protein and energy metabolism-related indicators in lambs.

Item	Time	CN	CLA-1	CLA-2	CLA-3	*p*-Value		
						Group	Time	Interaction
AST/(U/L)	d_7_	74 ± 6	70 ± 6	69 ± 6	64 ± 6	0.508	0.648	0.469
	d_42_	77 ± 5	74 ± 5	66 ± 5	66 ± 5			
β-HB/(mmol/L)	d_7_	0.34 ± 0.06	0.31 ± 0.06	0.30 ± 0.06	0.19 ± 0.06	0.65	0.117	0.215
	d_42_	0.43 ± 0.07	0.40 ± 0.07	0.33 ± 0.07	0.28 ± 0.07			
GLU/(mmol/L)	d_7_	8.92 ± 0.54 ^A^	8.71 ± 0.54 ^A^	8.40 ± 0.54 ^A^	7.81 ± 0.54 ^A^	0.522	0.002	0.606
	d_42_	7.24 ± 0.58 ^B^	7.22 ± 0.58 ^B^	6.97 ± 0.58 ^B^	6.30 ± 0.58 ^B^			
TG/(mmol/L)	d_7_	2.88 ± 0.41 ^Aa^	1.69 ± 0.41 ^Aab^	1.33 ± 0.41 ^Ab^	1.19 ± 0.41 ^Ab^	0.012	<0.001	0.137
	d_42_	1.05 ± 0.11 ^Ba^	0.79 ± 0.11 ^Bb^	0.74 ± 0.11 ^Bb^	0.73 ± 0.11 ^Bab^			
NEFA/(mmol/L)	d_7_	0.54 ± 0.12	0.52 ± 0.12	0.44 ± 0.12	0.40 ± 0.12	0.608	0.667	0.879
	d_42_	0.50 ± 0.07	0.47 ± 0.07	0.45 ± 0.07	0.36 ± 0.07			

Note: Different lowercase letters in the same row indicate significant differences among treatment groups at the same time point (*p* < 0.05). Different uppercase letters in the same column indicate significant differences between time points within the same treatment group (*p* < 0.05). The same letter or no letter indicates no significant difference (*p* > 0.05). Uppercase and lowercase letters are independent. P-group, P-time, and P-interaction denote the main effects of treatment group, time, and group × time interaction, respectively.

**Table 7:** Effects of conjugated linoleic acid on serum hormones in lambs.

Item	Time	CN	CLA-1	CLA-2	CLA-3	*p*-Value		
						Group	Time	Interaction
GH/(ng/mL)	d_7_	11.64 ± 0.77 ^Aa^	9.82 ± 0.77 ^Ba^	7.25 ± 0.77 ^Bb^	5.90 ± 0.77 ^Bc^	<0.001	0.033	0.376
	d_42_	11.54 ± 0.57 ^Ba^	10.66 ± 0.57 ^Aa^	9.71 ± 0.57 ^Ab^	7.52 ± 0.57 ^Ac^			
IGF-1/(ng/mL)	d_7_	1280 ± 80 ^b^	1500 ± 80 ^ab^	1440 ± 80 ^ab^	1810 ± 80 ^a^	<0.001	0.099	0.597
	d_42_	1340 ± 70 ^b^	1510 ± 70 ^ab^	1650 ± 70 ^ab^	1890 ± 70 ^a^			
GH/IGF-1 (×10^−3^)	d_7_	9.13 ± 0.60 ^a^	6.66 ± 0.60 ^ab^	5.13 ± 0.60 ^b^	3.31 ± 0.60 ^c^	<0.001	0.332	0.662
	d_42_	8.66 ± 0.53 ^a^	7.21 ± 0.53 ^ab^	5.97 ± 0.53 ^b^	3.99 ± 0.53 ^c^			

Note: Different lowercase letters in the same row indicate significant differences among treatment groups at the same time point (*p* < 0.05). Different uppercase letters in the same column indicate significant differences between time points within the same treatment group (*p* < 0.05). The same letter or no letter indicates no significant difference (*p* > 0.05). Uppercase and lowercase letters are independent. P-group, P-time, and P-interaction denote the main effects of treatment group, time, and group × time interaction, respectively.

**Table 8:** Effects of conjugated linoleic acid on serum antioxidant indices in lambs.

Item	Time	CN	CLA-1	CLA-2	CLA-3	*p*-Value		
						Group	Time	Interaction
SOD/(U/mL)	d_7_	2.08 ± 0.43	2.37 ± 0.43	2.73 ± 0.43	2.76 ± 0.43	0.315	0.902	0.678
	d_42_	2.18 ± 0.19	2.21 ± 0.19	2.59 ± 0.19	2.84 ± 0.19			
GPX/(U/mL)	d_7_	41.1 ± 2.8 ^B^	43.5 ± 2.8 ^B^	47.2 ± 2.8 ^B^	58.2 ± 2.8 ^B^	0.268	0.001	0.93
	d_42_	62.4 ± 9.6 ^A^	65.1 ± 9.6 ^A^	67.2 ± 9.6 ^A^	73.4 ± 9.6 ^A^			
MDA/(nmol/mL)	d_7_	1.23 ± 0.09 ^B^	1.22 ± 0.09 ^B^	1.12 ± 0.09 ^B^	1.16 ± 0.09 ^B^	0.247	<0.001	0.147
	d_42_	3.74 ± 0.49 ^A^	3.13 ± 0.49 ^A^	2.74 ± 0.49 ^A^	2.21 ± 0.49 ^A^			
T-AOC/(U/mL)	d_7_	0.19 ± 0.07 ^Ab^	0.28 ± 0.07 ^Ab^	0.56 ± 0.07 ^Aa^	0.60 ± 0.07 ^Aa^	<0.001	0.008	0.004
	d_42_	0.27 ± 0.03 ^Aa^	0.29 ± 0.03 ^Aa^	0.30 ± 0.03 ^Ba^	0.33 ± 0.03 ^Ba^			

Note: Different lowercase letters in the same row indicate significant differences among treatment groups at the same time point (*p* < 0.05). Different uppercase letters in the same column indicate significant differences between time points within the same treatment group (*p* < 0.05). The same letter or no letter indicates no significant difference (*p* > 0.05). Uppercase and lowercase letters are independent. P-group, P-time, and P-interaction denote the main effects of treatment group, time, and group × time interaction, respectively.

**Table 9:** Effects of conjugated linoleic acid on serum immune indicators in lambs.

Item	Time	CN	CLA-1	CLA-2	CLA-3	*p*-Value		
						Group	Time	Interaction
TNF-α/(ng/g)	d_7_	152 ± 7 ^a^	141 ± 7 ^a^	114 ± 7 ^b^	120 ± 7 ^b^	<0.001	0.553	0.483
	d_42_	147 ± 7 ^a^	141 ± 7 ^a^	123 ± 7 ^b^	104 ± 7 ^b^			
IL-8/(ng/g)	d_7_	581 ± 26 ^a^	490 ± 26 ^b^	447 ± 26 ^bc^	358 ± 26 ^c^	<0.001	0.072	0.604
	d_42_	569 ± 27 ^a^	433 ± 27 ^b^	382 ± 27 ^bc^	350 ± 27 ^c^			
IL-6/(ng/g)	d_7_	126 ± 7 ^Aa^	120 ± 7 ^Aab^	107 ± 7 ^Ab^	92 ± 7 ^Ac^	<0.001	0.011	0.439
	d_42_	124 ± 5 ^Ba^	102 ± 5 ^Bab^	98 ± 5 ^Bb^	74 ± 5 ^Bc^			
IL-1β/(ng/g)	d_7_	66.7 ± 2.7 ^Aa^	61.1 ± 2.7 ^Aab^	50.2 ± 2.7 ^Ab^	45.1 ± 2.7 ^Ab^	<0.001	<0.001	0.618
	d_42_	57.0 ± 2.3 ^Ba^	49.7 ± 2.3 ^Bab^	45.1 ± 2.3 ^Bb^	37.7 ± 2.3 ^Bb^			

Note: Different lowercase letters in the same row indicate significant differences among treatment groups at the same time point (*p* < 0.05). Different uppercase letters in the same column indicate significant differences between time points within the same treatment group (*p* < 0.05). The same letter or no letter indicates no significant difference (*p* > 0.05). Uppercase and lowercase letters are independent. *P*-group, *P*-time, and *P*-interaction denote the main effects of treatment group, time, and group × time interaction, respectively.

## Data Availability

The datasets generated during and/or analyzed during the current study are available from the corresponding author upon reasonable request.

## Abbreviations

β-HB = β-hydroxybutyrate; CLA = conjugated linoleic acid; GH = growth hormone; IGF-1 = insulin-like growth factor-1; MDA = malondialdehyde; NEB = negative energy balance; NEFA = nonesterified fatty acids; SOD = superoxide dismutase; T-AOC = total antioxidant capacity; TG = triglycerides; TNF-α = tumor necrosis factor-α.
